# Supplementary material for: Viral-inducible Argonaute18 confers broad-spectrum virus resistance in rice by sequestering a host microRNA
Source: eLife. 2015 Feb 17;4:e05733. doi: 10.7554/eLife.05733 (PMC4358150; doi:10.7554/eLife.05733)
Supplement: Supplementary file 2. — Perl script used for statistical analysis of miRNA expression. DOI: http://dx.doi.org/10.7554/eLife.05733.016 [file elife05733s002.docx]

**Supplementary file 2.** Perl script used for statistical analysis of miRNA expression.

#!/usr/bin/perl

use strict;

#this perl script accepts small RNA alignment files as input, the output is the RPM of each annotated miRNA expression. Arguments as belows:

#overlap: the overlapped file between mapped bam file of small RNAs and annotated miRNA coordination (generated by intersectBed)

#total: the total number of mapped small RNA reads

#bam: mapped bam file of small RNAs

#miRNA: annotated miRNA coordination

# Perl scripts by Qi Lab, Tsinghua University, Beijing, China

die "perl $0 overlap total bam miRNA\n" unless (@ARGV==4);

my ($overlap,$total,$bam,$miRNA)=@ARGV;

my %hash;

use File::Basename;

my ($name)=basename($bam)=~/(.*)\.bam/g;

my %hash1;

my $RPM;

open (IM,$total);

while(<IM>){

chomp;

my @array=split;

$hash1{$array[0]}=$array[2];

}

open (IN, $overlap);

while(<IN>){

chomp;

my @str=split;

my ($abu)=$str[3]=~/.*_.*_(.*)/g;

print "$abu\n";

$hash{$str[-4]}+=$abu;

}

open (FILE,$miRNA);

while(<FILE>){

chomp;

my @block=split;

if (defined($hash{$block[3]})){

$RPM=$hash{$block[3]}/$hash1{$name}*1000000;

print "$block[3]\t$block[0]\t$block[1]\t$block[2]\t$hash{$block[3]}\t$RPM\n";

} else {

print "$block[3]\t$block[0]\t$block[1]\t$block[2]\t0\t0\n";

}

}
